# Unilateral pulmonary vein atresia presenting with recurrent hemoptysis and bronchial varices in an Ethiopian adolescent: a case report

**DOI:** 10.1186/s13256-023-03956-4

**Published:** 2023-06-03

**Authors:** Abate Yeshidinber Weldetsadik, Abdi Kebede, Binyam Gebremedhin Godu, Maru Gama

**Affiliations:** 1 Department of Pediatrics and Child Health, Saint Paul’s Hospital Millennium Medical College, Addis Ababa, Ethiopia; 2 Pediatric Surgery Unit, Saint Paul’s Hospital Millennium Medical College, Addis Ababa, Ethiopia; 3 Department of Surgery, Pediatric Surgery Unit, Saint Paul’s Hospital Millennium Medical College, Addis Ababa, Ethiopia

**Keywords:** Pulmonary vein atresia, Recurrent hemoptysis, Chest infection, Case report, Ethiopia

## Abstract

**Background:**

Congenital unilateral pulmonary vein atresia is a rare anomaly resulting from failure of the pulmonary vein to incorporate in the left atrium. It is a very rare cause of recurrent respiratory infections and hemoptysis requiring a high index of suspicion for proper diagnosis and management in early childhood.

**Case presentation:**

We report a 13-year old Anuac (Ethiopia, Region of Gambela) male adolescent with a delayed diagnosis of isolated atresia of the left pulmonary veins despite early childhood presentation with recurrent chest infections, hemoptysis and exercise intolerance. Contrast enhanced CT of thorax with reconstructed planes confirmed the diagnosis. He underwent pneumonectomy for severe and recurrent symptoms and did well on subsequent follow ups after 6 months of pneumonectomy.

**Conclusion:**

Although a rare anomaly, congenital unilateral pulmonary vein atresia should be considered in the differential diagnosis of a child presenting with recurrent chest infections, exercise intolerance and hemoptysis to facilitate early appropriate diagnosis and treatment.

## Background

Isolated Congenital Unilateral Pulmonary Vein Atresia (CUPVA) is a rare anomaly that results from failure of the pulmonary vein to incorporate in to the left atrium during embryogenesis [1–4]. As it is a very rare cause of recurrent chest infections and hemoptysis, a high index of suspicion is required for the proper diagnosis and management of CUPVA in early childhood [2–6]. While majority of CUPVA patients present in early life, mostly in the first 3 years of age, diagnosis may be delayed to adolescence and adulthood because of presence of comorbid conditions or missed diagnosis, patients may remain asymptomatic or failure to consider CUPVA in the differential as in our patient [2–8].

Failure of incorporation of the pulmonary veins into the left atrium results in persistence of connections between the pulmonary circulation and the systemic veins that result in the presence of mediastinal collateral vessels. These collaterals may appear as a mediastinal mass on chest CT [9–11].

Our case is of interest to others generally because of the very rare occurrence of CUPVA, and the late diagnosis despite early childhood symptomatology. We thus report this case to highlight the need for clinicians to consider CUPVA as a differential in children with recurrent hemoptysis and repeated respiratory infections.

## Case presentation

A 13-year old Anuac (Ethiopia, Region of Gambela) male adolescent was referred to our pulmonary clinic for recurrent hemoptysis and repeated respiratory infections. He started to expectorate bright bloody sputum, sometimes as much as 30–50 ml per day, intermittently starting from 4 years of age with gradually increasing hemoptysis especially in the last 3 weeks before presentation. He received treatment with multiple courses of unspecified oral antibiotics for up to 5–10 days for recurrent episodes of cough and fever with shortness of breath since infancy; and he gradually developed worsening exercise intolerance with dyspnea on exertion, palpitation and non-radiating left side dull aching chest pain especially in the last 5 years.

These symptoms occur 4–5 times in a year for which he was prescribed oral antibiotics which improved symptoms for few months. He was treated for pulmonary tuberculosis with Rifampicin, Isoniazid, Pyrazinamide and Ethambutol combination for 2 months followed by Rifampicin and Isoniazid of 4 months at the age of 6 years with clinical suspicion and left hemithorax opacities on chest x-ray but negative microbiologic studies from gastric aspirate and lymph node cytology. Otherwise, he had optimal developmental history and he was a grade 7 student with average school performance. Apart from those mentioned above, his past medical, social, environmental, and family history were unremarkable.

On examination, he was healthy looking with BP of 100/65 mmHg, PR of 100, axillary temperature of 36.4 degree Celsius and oxygen saturation of 94% in room air. He weighed 36 kg, height of 160 cm (BMI; between –2 and –3 SD; other indices above –2 SD). Chest examination revealed decreased air entry on left hemithorax with relative dullness. Head and neck, lymphoglandular, cardiac, abdomen, genitourinary, musculoskeletal and neurologic exams were all normal at presentation.

**Fig. 1:**
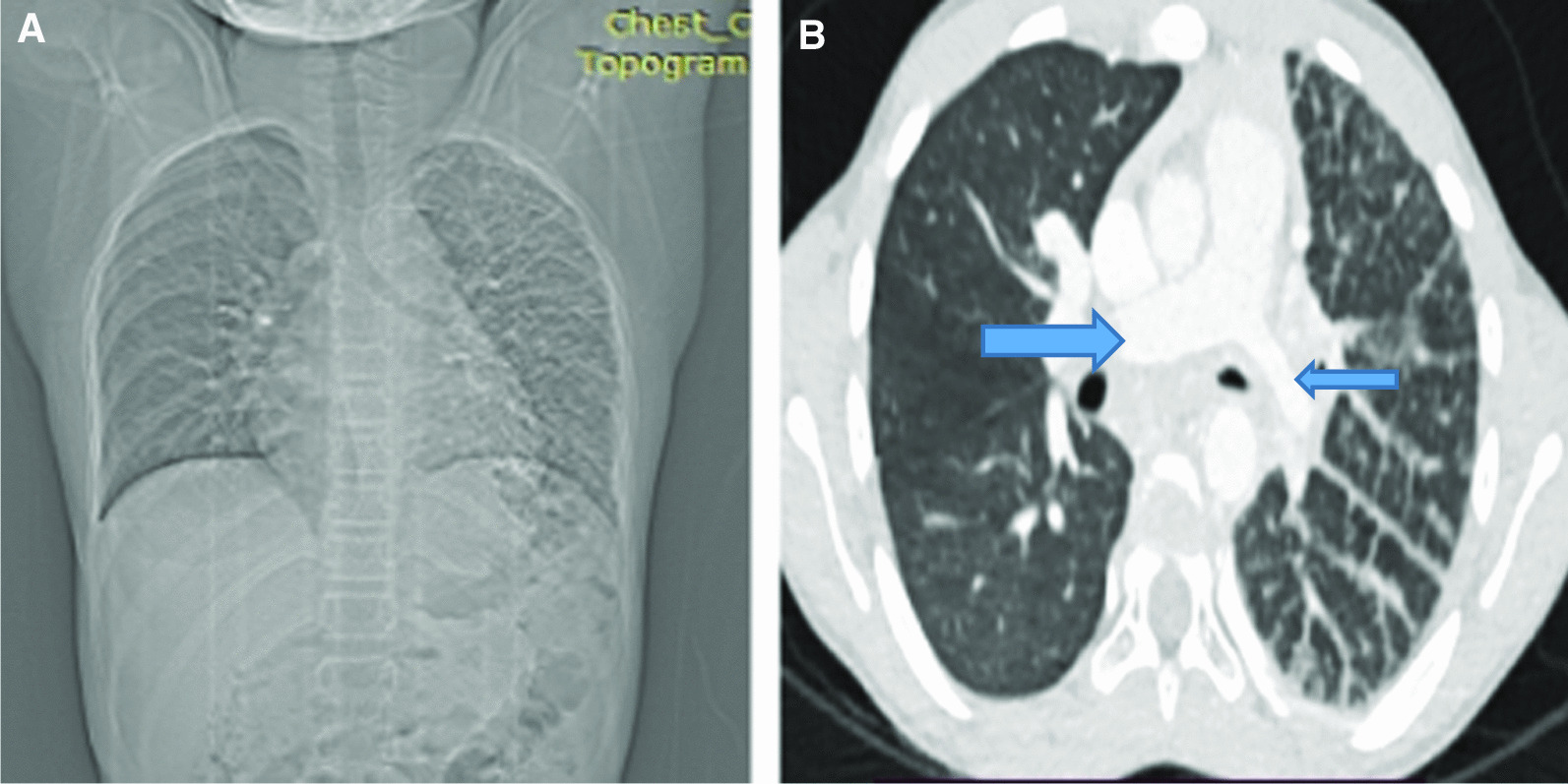
**A** Postero Anterior chest x-ray (PA CXR). Left hemithorax appears smaller and diffuse reticulation with smaller hilum. Right hemithorax is normal. **B** Contrast enhanced chest Computed tomography showing left lung diffuse interlobular septal thickening and ground glass opacity with a normal right lung. Note the significantly smaller left pulmonary artery (smaller arrow) as compared to the right (bigger arrow)

**Fig. 2:**
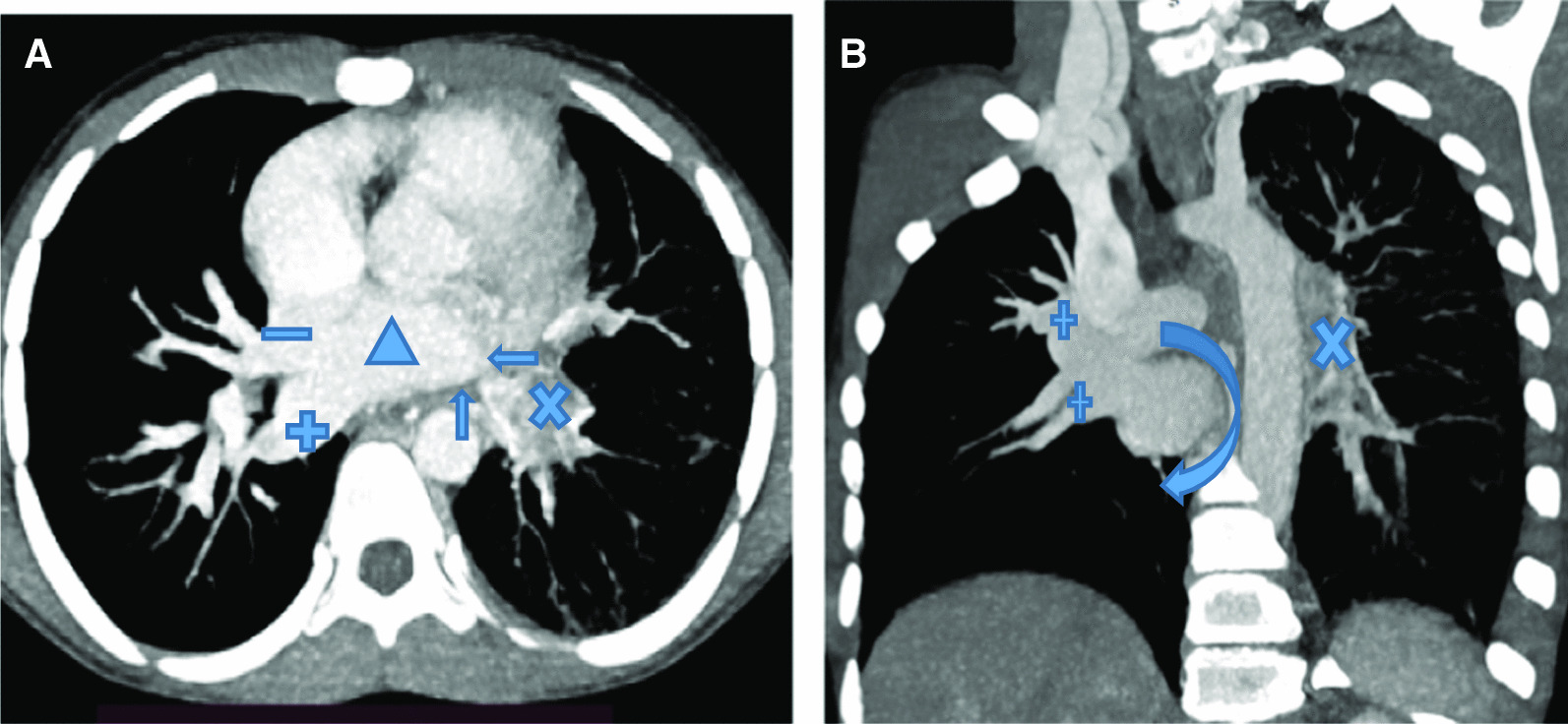
**A** Axial view Computed tomography angiography showing Left Atrium (∆), right upper (−) and right lower ( +) pulmonary veins. Left boarder of left atrium is smooth (arrows). Vascular anastomosis appears as a left hilar ill-defined mass (x). **B** Left posterior corona image of Computed tomography angiography with smooth Left Atrium (curved arrow) on the left and branching pulmonary veins on the right ( +). Left hilar vascular anastomosis (x) also seen

Complete blood count (WBC = 7800, N = 56%, L = 42%, Hemoglobin of 13.5 mg/dl, Platelets = 322,000/ul) and acute phase reactants (ESR = 5, CRP was negative) were normal and screening for tuberculosis was negative. Chest radiograph (Fig. 1A) showed smaller left lung with hilum not well visualized and diffuse reticulation; and a normal right hemithorax. A contrast enhanced chest Computed Tomography (CT) (Fig. 1B) showed diffuse ill-defined mass in the mediastinum and left hilum bounding the left pulmonary artery and left bronchus and its branches (not shown here); and diffuse left lung inter-lobular septal thickening, and right lung was normal. The left pulmonary artery was smaller than the right and the left pulmonary veins were not visualized on Contrast enhanced coronal and reconstructed planes (Figs. 2A, B).

**Fig. 3:**
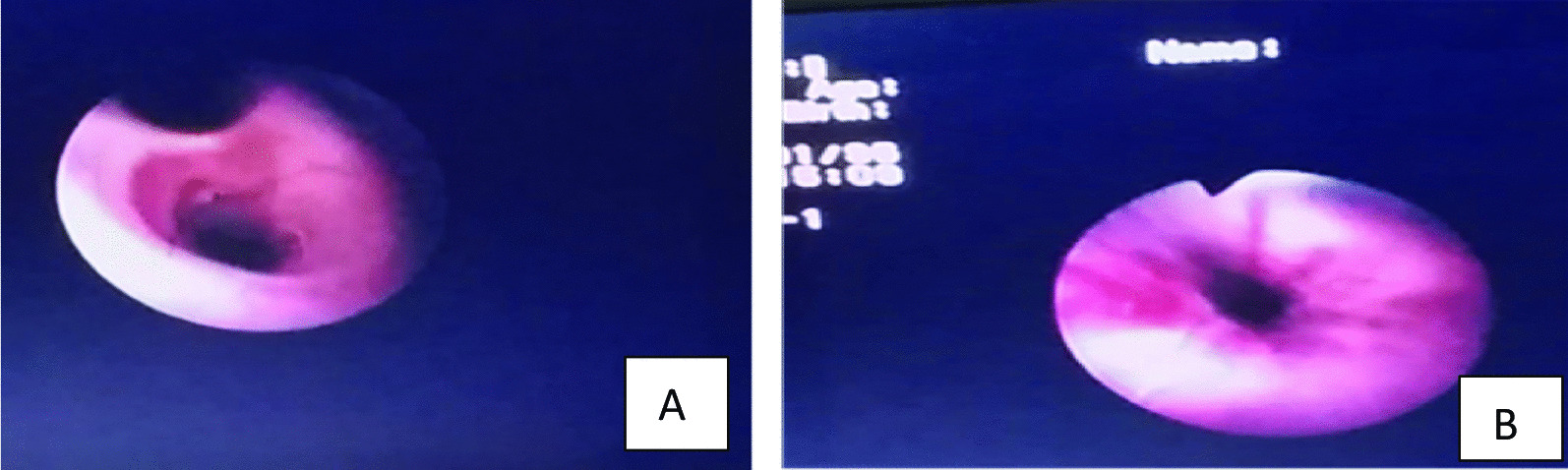
Bronchoscopic view of the normal right main bronchus (**A**) and the left main bronchus with bronchial Varices (**B**). The left bronchus and its divisions were hyperemic with numerous tortuous mucosal veins that easily bleed on touch and the mucosal wall was also edematous and narrowed.

Flexible bronchoscopy demonstrated edematous mucosa of the left bronchial divisions with increased vascularity and multiple bronchial varices starting from the main bronchus and extends distally to all left lobar bronchi (Fig. 3). Echocardiography reported decreased size of the left atrium but no other structural heart defected or pulmonary hypertension. Ventilation and perfusion scan and cardiac catheterization were not available in our setting. After the child was evaluated by a team of pediatricians and pediatric surgeons, pneumonectomy was decided because of severe and recurrent symptoms. Endovascular stent implantation was not done because of delayed diagnosis, limited experience in our setting with additional problem of device availability.

Left posterolateral thoracotomy revealed heavy and fleshy appearance of the affected left lung. The pulmonary artery was judged normal but the left pulmonary viens were rudimentary. There were no enlarged hilar lymph nodes but the left lung was adhered to the left lateral chest wall and diaphragm. Left pneumonectomy was done after releasing the adhesion with minimal intraoperative bleeding. He received 2 doses of (prophylactic) ceftriaxone at 50 mg/kg IV 30 min before and 12 h after surgery based on the hospital protocol. He has also received combination of analgesics after thoracotomy including diclofenac 1 mg/kg IM PRN and morphine orally as needed. The post-operative course was smooth, chest drain removed on day 4 and patient discharged stable on sixth post-operative day. The child recovered uneventfully from the surgery and remains asymptomatic after 6 months of follow up post-pneumonectomy. On the follow up, he reported normal activity with no limitation, has restarted school and has no scoliosis, chest pain or cough. A control CXR showed complete opacification of the left hemithorax with no mediastinal shift and normal right lung with hyperinflation.

## Discussion

In this case report, a 13-year old male with early onset hemoptysis and recurrent respiratory infection was lately diagnosed to have CUPVA using contrast enhanced CT of thorax with reconstructed planes and bronchoscopy demonstrating bronchial varices. The child underwent pneumonectomy and did well on subsequent follow ups up to 6 months postpneumonectomy.

CUPVA is an extremely rare malformation with nearly 50 cases reported till 2018 [3] and just more than 12 case then after [4, 11–15]. While congenital heart diseases may be found in up to 50% of patients, isolated CUPVA as in our patient is even more rare [3].

The most frequent manifestations of CUPVA in infancy include recurrent infections in the hypoplastic lung and hemoptysis. The increased risk of infection has various factors related to a hypoplastic lung, including impaired mucocilliary clearance, impaired local immunity, and impaired venous and lymphatic drainage. The typical radiographic features of CUPVA include a small ipsilateral hemithorax with reticular opacities and septal lines, pleural thickening or effusion, ipsilateral mediastinal shift, a small ipsilateral pulmonary artery, and absence of pulmonary venous drainage into the left atrium and ipsilateral mediastinal mass [1–11]. Reticular opacities in the smaller lung corresponds to thickened interlobular septae likely from chronic changes of venous hypertension and impaired lymphatic drainage while the decreased lung volume may be related to preferential arterial perfusion of the normal lung, impaired growth in the ipsilateral pulmonary artery, and chronic parenchymal scarring and changes [1, 4]. Rupture of dilated bronchial veins (varices) as a result of pulmonary venous obstruction is the main cause of hemoptysis [6, 7].

Diagnosis of CUPVA is challenging and should be differentiated from other causes of diffuse unilateral pulmonary diseases such as bronchiectasis, radiation pneumonitis, pulmonary hypoplasia (primary/secondary), poor blood flow to the lung (Scimitar syndrome and Swyer-James syndrome), and acquired pulmonary obstruction (mediastinal tumors, fibrosing mediastinitis and pulmonary veno-occlusive disease) [8, 12, 13]. Presence of smaller affected lung with diffuse interlobular septal thickening and ipsilateral small pulmonary artery differentiate CUPVA from those other causes [13]. CT angiography findings of a small ipsilateral pulmonary artery with absence of venous opacification on venous phase are diagnostic. The presence of bronchial varices is a strong additional evidence to diagnose CUPVA in a patient with the above radiologic signs ([3, 6, 7, 13]. Cardiac catheterization remains gold standard for diagnosis of CUPVA in many countries. However, cardiac catheterization can be avoided in cases where diagnosis is confirmed on imaging studies [8, 10, 14]. Ventilation perfusion scans confirm reduced perfusion to the affected lung, with large mismatch and increased dead space [9].

The optimal management of CUPVA is not yet known because of its rarity, with data being available only from case reports [2–4]. Indication for treatment is based on risk of life-threatening hemoptysis, recurrent infections, and development of pulmonary hypertension. Asymptomatic patients can be followed closely without surgical intervention [8]. Treatment of symptomatic CUPVA patients usually requires surgical interventions, and recurrent infections, hemoptysis and pulmonary hypertension are the main reasons for surgery [2, 11]. A variety of conservative surgeries are available for restoring continuity between atretic pulmonary veins and the left atrium if diagnosis is made in early life [2]. However, as the majority of patients present after irreversible changes have occurred in the pulmonary parenchyma and vasculature, reconstitution of flow to left atrium does not allow restoration of pulmonary function and remodeling of the pulmonary vasculature [2, 4]. Pneumonectomy is the ultimate surgical treatment, which will not only remove the nidus for infections and hemoptysis but also relieve the significant left-to-right shunt and the increased dead space that causes exercise limitation and hypoxia. The prognosis following pneumonectomy is favorable, with good functional results [2]).

## Conclusion

We discussed a case of an adolescent with recurrent hemoptysis and respiratory infections diagnosed with isolated unilateral atresia of the pulmonary vein. Though CUPVA is a rare condition, it should be considered in the differential diagnosis of a child with recurrent chest infections and hemoptysis. Diagnosis can be made based on appropriate history and examination, chest X-ray, echocardiography and chest CT angiography without invasive diagnostic studies. Therapeutic strategies for CUPVA are mainly guided by the presence of cardiac and respiratory symptoms and /or complications. The decision to perform pneumonectomy is based on the severity of complications, including hemoptysis, recurrent infections, and pulmonary hypertension.

## Data Availability

Further information on the case including diagnostic and therapeutic details can be made available from the corresponding author on request.

## Abbreviations

BMI: Body mass index
CT: Computed tomography
CUPVA: Congenital unilateral pulmonary vein atresia
SD: Standard deviation
